# Origins of two hemiclonal hybrids among three *Hexagrammos* species (Teleostei: Hexagrammidae): genetic diversification through host switching

**DOI:** 10.1002/ece3.2446

**Published:** 2016-09-14

**Authors:** Hiroyuki Munehara, Miho Horita, Motoko R. Kimura‐Kawaguchi, Aya Yamazaki

**Affiliations:** ^1^ Field Science Center for Northern Biosphere Hokkaido University Hakodate Hokkaido Japan; ^2^ Graduate School of Environmental Science Hokkaido University Hakodate Hokkaido Japan; ^3^ Division of Analytical Bio‐Medicine Advanced Research Support Center Ehime University Toon City Ehime Japan

**Keywords:** hybridogenesis, diversification of hybrids, host switching, maternal inheritance, Improving longevity through host switching backcross

## Abstract

Two natural, hemiclonal hybrid strains were discovered in three *Hexagrammos* species. The natural hybrids, all of which were females that produced haploid eggs containing only the *Hexagrammos octogrammus* genome (maternal ancestor; hereafter *Hoc*), generated F_1_ hybrid‐type offspring by fertilization with haploid sperm of *Hexagrammos agrammus* or *Hexagrammos otakii* (paternal species; *Hag* and *Hot*, respectively). This study was performed to clarify the extent of diversification between the two hybrids and the maternal ancestor. Genealogical analysis using mtDNA revealed that all 38 *Hoc*/*Hot* hybrids formed a branch (Branch I) with 18 of the 33 *Hoc*/*Hag* hybrids. No haplotype sharing was observed with the maternal ancestor. Further, microsatellite DNA analysis suggested that the members of Branch I shared the same hemiclonal genome set. The results suggested that *Hoc*/*Hot* hybrids originated by anomalous hybridization, or “host switching,” between *Hoc*/*Hag* and *Hot*, and not from interspecific hybridization between *Hoc* and *Hot*. The remaining 9 of 11 *Hoc*/*Hag* haplotypes and all of the 27 *Hoc* haplotypes were mixed within the genealogical tree, as if they had originated from multiple mutations. However, *Hoc*/*Hag* could also mate with *Hoc*. Although offspring from this host switch (Backcross‐*Hoc*) have the same genome as normal *Hoc*, a part of their genome retains genetic factors capable of producing hemiclones. Consequently, when a descendant of a BC‐*Hoc* hybrid mates with *Hag* males, a new hemiclone lineage will arise. Multiple haplotype revival through host switching from a single mutation in hybrids is another possible hypothesis for the observed mixing of *Hoc*/*Hag* haplotypes within the mtDNA genealogical tree.

## 1 Introduction

Although most eukaryotes have retained sexual reproduction with recombination as a reproductive strategy, some have developed unisexual modes of reproduction, such as clonal reproduction (parthenogenesis and gynogenesis) and hemiclonal reproduction (hybridogenesis), which involve hybridization between different species (Dawley, 1989; Hubbs & Hubbs, 1932; Lampert & Schartl, 2008). In clonal modes of unisexual reproduction, females produce unreduced diploid or triploid eggs by different cytogenetic mechanisms that develop normally without any biological or genetic contribution from males, and no male offspring are produced (Dawley, 1989). Unlike sexual reproduction, in which there is the added cost of producing of males, unisexually reproducing organisms do not incur these additional costs during reproduction (Maynard Smith, 1978). Consequently, unisexual taxa are considered to be at an advantage in terms of their ability to colonize new habitats and outcompete organisms that employ sexual reproduction (Avise, 2008). However, unlike sexually reproducing organisms, unisexual taxa are more likely to accumulate deleterious mutations (Kondrashov, 1988; Rice & Friberg, 2009). Consequently, the long‐term survival of unisexual taxa that lack novel genetic adaptations to perturbations in the environment or to attacks by parasites is relatively limited (Bengtsson, 2009; Neiman & Koskella, 2009), implying that they are potentially evolutionary dead ends that are at greater risk of extinction (Bell, 1982; Bengtsson, 2009; Maynard Smith, 1986). In response to these limitations, several mechanisms have been identified that mitigate against the severe genetic disadvantages associated with a unisexual mode of reproduction (Loewe & Lamatsch, 2008; Schartl, Wilde, Schlupp, & Parzefall, 1995). For example, in the Amazon molly (*Poecilia formosa*), small parts of the paternal genome (microchromosomes) can remain in the oocyte during gynogenesis (Lamatsch, Nanda, Schlupp, Epplen, & Schmid, 2004; Schartl et al., 1995). In addition, polyploidy has been observed in both clonal and hemiclonal species, including the topminnow (*Poeciliopsis monacha‐lucida*) (Cimino & Schultz, 1970; Schultz, 1969; Vrijenhoek, Dawley, Cole, & Bogart, 1989), *Pelophylax* (formerly *Rana*) water frog complex (Graf & Pelaz, 1989), *Cobitis* spiny loach hybrids (Choleva et al., 2012; Janko, Culling, Ráb, & Kotlík, 2005; Janko et al., 2007), and the Pond loach (*Misgurnus anguillicaudatus*) (Itono et al., 2006). These additional genome components can benefit an organism by providing more genetic material that can be acted on by mutation and selection (Ohno, 1970; Volff 2005). The presence of these additional genetic materials have enabled some unisexual organisms to mitigate against the effects of deleterious mutations (Loewe & Lamatsch, 2008; Schartl, Nanda, et al., 1995), enabling unisexual systems to persist and last for longer than predicted periods over evolutionary time (Loewe & Lamatsch, 2008; Lynch & Gabriel, 1990; Maynard Smith, 1992). For example, the Amazon molly has been estimated to be about 120,000–280,000 years old, which is equivalent to approximately 360,000–840,000 generations (Lampert & Schartl, 2008; Meyer, Salzburger, & Schartl, 2006; Schartl, Wilde, et al., 1995; Stöck, Lampert, Möller, Schlupp, & Schartl, 2010), and *Cobitis* spiny loach hybrids have existed for approximately 300,000 years (Janko et al., 2005).

Hybridogenesis is a form of hemiclonal reproduction that results in the production of haploid eggs that contain only the maternal genome as the paternal genome is discarded (Cimino, 1972; Schultz 1969, Schultz, 1973). Interestingly, the females reproduce by backcrossing with males of the paternal ancestor; this phenomenon has been reported in the topminnow (*P. monacha‐lucida*), *Pelophylax* water frog complex (Ogielska, 2009; Uzzell & Berger, 1975), stick insects (Scali, 2009), Iberian minnow (Carmona, Sanjur, Doadrio, Machordom, & Vrijenhoek, 1997), Australian carp gudgeon (*Hypseleotris* hybrid) (Schmidt, Bond, Adams, & Hughes, 2011), and greenling (*Hexagrammos* hybrid) (Kimura‐Kawaguchi et al., 2014). Male hybridogenesis in which the maternal genome is discarded has also been reported in the *Pelophylax* water frog complex (Lehtonen, Schmidt, Heubel, & Kokko, 2013) and the Australian carp gudgeon (Schmidt et al., 2011).

In hybridogenesis, although females produce genetically identical haploid eggs without any genetic recombination, genetic variation is maintained by renewal of the paternal genome every generation. In this way, hybridogenesis compensates for the costs associated with sexual reproduction while retaining some of the benefits of clonal reproduction (Vrijenhoek, 1994). These advantages, despite involving a unisexual mode of reproduction, should enable hemiclonal animal lineages to remain viable for longer than clonal lineages.

Hybridogenesis, gynogenesis, and parthenogenesis are all considered to have originated from hybridization between different species (Lamatsch & Stöck, 2009; Vrijenhoek, Angus, & Schultz, 1977). Speciation in two geographically separated populations can occur when a contiguous population is separated by a vicariant event of some kind. Under such conditions, genetic differences gradually arise between the separated populations, often resulting in what is referred to as allopatric speciation (Coyne & Orr, 2004). In such cases, if secondary contact occurs before premating reproductive isolation has fully developed, natural hybrids will appear (Barton & Hewitt, 1989). Although most hybrids typically have low fitness and low reproductive viability due to the inherent incompatibility of the genomes from different species, in some instances, hybrids may be able to survive by employing unisexual reproduction without the recombination of genomes (Ellstrand et al., 2010).

For example, hemiclonal reproduction has recently been reported in two *Hexagrammos* hybrid strains (Kimura‐Kawaguchi et al., 2014). The natural hybrids produce haploid eggs containing only the *Hexagrammos octogrammus* genome (maternal ancestor) and generate F_1_ hybrid‐type offspring by fertilization with the haploid sperm of either *Hexagrammos agrammus* or *Hexagrammos otakii* (paternal species); in this way, the genome set of the natural hybrids is composed of a hemiclonally transmitted maternal genome and a recombined paternal genome. Similarly, because the second generations of a backcross between natural hybrids and paternal species reproduce by hybridogenesis in the same way as the maternal generation of the natural hybrids, hemiclonal reproduction is maternally inherited over successive generations by backcrossing with paternal species. In addition, Kimura‐Kawaguchi et al. (2014) also found that artificial F_1_ hybrids produced by crossing pure species generated recombinant gametes, suggesting that although the artificial F_1_ hybrids have the same genome composition as hemiclonal hybrids, hemiclonal hybrids do not always result from a hybridization event. In addition, hemiclonal hybrids exhibit genetic differences (mutations) that do not occur in wild‐type parental species.

Maternal inheritance markers can be used to clarify when hybridization occurred. Genealogical relationships among parental Hexagrammid species were estimated using polymorphic mitochondrial and nuclear DNA markers (Crow, KAnamoto, & Bernardi, 2004). *Hexagrammos agrammus* and *H. otakii* (paternal species) are the most closely related taxa in this genus. The common ancestor of *H. agrammus* and *H. otakii* (paternal species) underwent allopatric divergence from *H. octogrammus* (maternal ancestor) approximately 2.2–3.6 million years ago, and *H. otakii* and *H. agrammus* underwent sympatric divergence from the common ancestor approximately 1.2–2.0 million years ago. Secondary contact between the maternal and paternal species probably occurred after sympatric speciation during the Pleistocene (Brykov & Podlesnykh, 2001; Shinohara, 1994). *Hexagrammos* hybridization is the only known hybridogenetic system in marine fishes inhabiting the North Pacific Ocean. The potentially low extinction potential of these *Hexagrammos* hybrids is considered to be due to the diversity of habitats, and the longevity, structure, and fluctuation in populations of these species would likely differ from (hemi)clonal organisms distributed in more restricted environments, such as rivers and ponds. The present study was conducted to clarify the origin and diversification of two *Hexagrammos* hybrids and the maternal parent species (*H. octogrammus*) using maternal inheritance markers.

## 2 Materials and Methods

### 2.1 Fish sampling and species identification

**Figure 1:**
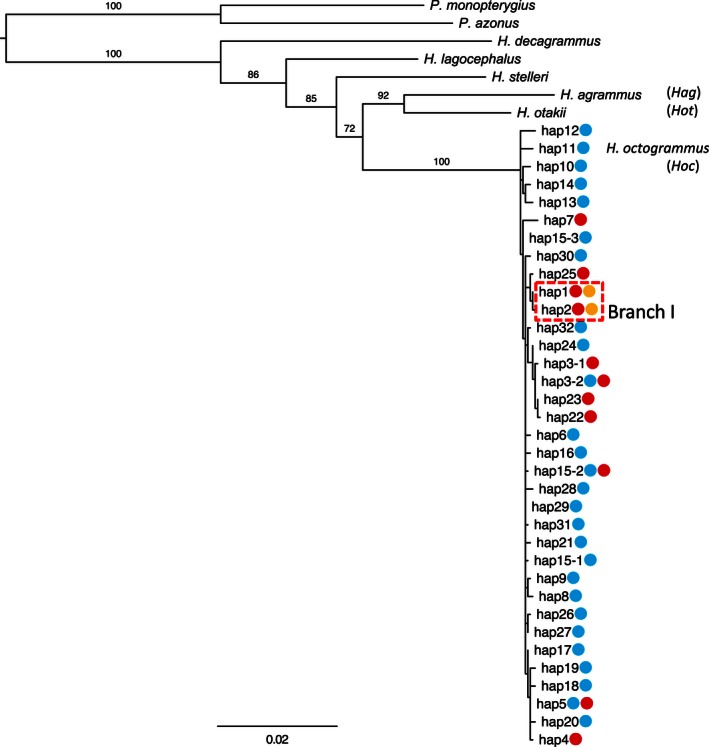
Genealogical tree for *Hoc*,* Hoc*/*Hag*, and *Hoc*/*Hot* derived from three regions of mtDNA:* Cyt* b, *12S‐16S rRNA*, and *CoI*. Numbers above nodes indicate bootstrap values obtained from 1000 replications. The colored symbols adjacent to each haplotype indicate the presence of *Hoc* (blue), *Hoc*/*Hag* (red), and *Hoc*/*Hot* (yellow)

For genealogical analysis of the two natural, hemiclonal, hybrid strains, *H. octogrammus*/*H. agrammus* (*Hoc*/*Hag*) and *H. octogrammus*/*H. otakii* (*Hoc*/*Hot*), and the maternal ancestor *H. octogrammus* (*Hoc*), fishes were captured using gill nets and traps on a coastal reef off Usujiri, Japan, from 2004 to 2010 (Fig. 1). Specimens were identified based on diagnostic external morphological diagnostic characteristics, such as the number of lateral lines, flap pairs, and the caudal fin shape, following Nakabo (2000) and Shinohara (1994), as described previously (Kimura‐Kawaguchi et al., 2014). A total of 40 *Hoc*, 31 *Hoc*/*Hag*, and 38 *Hoc*/*Hot* specimens were used in the present study. Muscle or fin tissue samples were collected from the fish and preserved in 99% ethanol at −10°C until genetic analysis. The paternal species, *H. agrammus* and *H. otakii*, and the closely related *Hexagrammos decagrammus*,* Hexagrammos lagocephalus*,* Hexagrammos stelleri*,* Pleurogrammus azonus*, and *Pleurogrammus monopterygius*, all of which are held in the collection at the Usujiri Fisheries Station, were included in the genealogical analysis.

In addition, to estimate the allele frequencies for the paternal species of the two natural hybrid strains using microsatellites, 33 *H. agrammus* and 34 *H. otakii* were captured using hand nets while SCUBA diving on a coastal reef off Usujiri, Japan, from 2010 to 2013. Tissues from these specimens were preserved in 99% ethanol and stored at −10°C until genetic analysis.

### 2.2 Polymerase chain reaction conditions and mitochondrial DNA sequencing

Total genomic DNA was extracted using a Quick Gene DNA tissue kit S (Fujifilm, Japan) according to the manufacturer's instructions and stored in a refrigerator at 4°C until use.

Three regions of the mitochondrial genome (i.e., cytochrome *b* [cyt *b*], 12S‐16S rRNA, and cytochrome oxidase [CO] I) of the mitochondrial DNA of *Hoc*/*Hag*,* Hoc*/*Hot*,* Hoc*, and the outgroup species were sequenced (Table 1). The first two regions and the third region were amplified using the primer sets of Kimura, Yanagimoto, and Munehara (2007) and Ward, Zemlak, Innes, Last, and Hebert (2005), respectively. Polymerase chain reactions (PCRs) were performed in 50 μl volumes containing 25 μl Emerald Amp™ PCR Master Mix (Takara Bio Inc., Japan), 22 μl sterile distilled water, 0.5 μl of each 5 μmol/l primer, and 2 μl of template DNA (50–100 ng). The PCR profiles for the three regions consisted of an initial denaturation step at 94°C for 2 min, followed by 30–40 cycles of denaturation at 94°C for 30 s, annealing at 55°C, and extension at 72°C for 30 s, with a final extension step of 72°C for 7 min. After the final extension step, samples were stored at 4°C. Amplification was performed using a Takara PCR Thermal Cycler Dice (Takara Bio Inc.), and PCR products were purified using a NucleoSpin^®^ Gel and PCR Clean‐up kit (Macherey‐Nagel GmbH & Co. KG, Germany). PCR products were sequenced with an autosequencer (3130 Genetic Analyzer, Applied Biosystems, CA) by Macrogen Japan Corporation using the same PCR primers.

**Table 1:** PCR primer sequences used in the present study

Locus (accession no.)	Sequence 5′‐‐‐‐‐‐‐‐‐‐‐3′ (upper, forward; lower, reverse)
*cytochrome b* (AF087409, 087410, 087412)	ATGGCAAGCCTACGAAAA
	TCCTAAGGCCTTGTTTTCT
*12‐16S rRNA* (AF084629, 084631)	CGGGAACTACGAGCAAAAG
	TCTTTTAGTCTTTCCCTGGGG
*COI* (DQ107581–DQ108334)	TCAACCAACCACAAAGACATTGGCAC
	TAGACTTCTGGGTGGCCAAAGAATCA
*hexoc 6* (AB690324)	GGATAGTTTGTTCCTGTCAG
	AAATGTTTGTCCCCAAACCC
*hexoc 14* (AB690329)	CGGGGTAGTGAAGCATGAT
	TTTTGTACTTGTGTTTTCCT
*hexoc 21* (AB690332)	CACATTTCTACAACAGCTTG
	AGTTATGACATGAGCTGAAGA

### 2.3 Sequence analysis

Specimen sequences were aligned using the Clustal W computer program (Higgins, Thompson, & Gibson, 1994). Genealogical analysis among haplotypes was performed using MEGA software (version 6.06; Tamura, Stecher, Peterson, Filipski, & Kumar, 2013). The nucleotide substitution model for each gene was selected using Kakusan4 (Tanabe, 2011), and sequence data were also subjected to a maximum‐likelihood (ML) analysis. Phylogenetic relationships between each partition were inferred by the ML method using RAxML (version 7.2.8; Stamatakis, 2006). Nucleotide divergences were computed using the Kimura 2‐parameter model (Kimura, 1980), and a genealogical tree was constructed using the neighbor‐joining method (Saitou & Nei, 1987). The robustness of the topology nodes was assessed using the bootstrap method with 1000 replications (Felsenstein, 1985). Unrooted statistical parsimony haplotype networks were created to connect mitochondrial DNA (mtDNA) haplotypes using TCS 1.21 (Clement, Posada, & Cradell, 2000).

### 2.4 Allelic analysis using microsatellite DNA

In hybridogenesis, nuclear DNA inherited from the maternal ancestor is maternally inherited in the same way as mtDNA, which means that microsatellite marker is also well suited for genealogical analysis in hemiclone organisms. To examine the sharing of alleles among the natural hybrids (*Hoc*/*Hag* and *Hoc*/*Hot*) and *Hoc*, three highly polymorphic microsatellite loci (*hexoc 6*,* hexoc 14*, and *hexoc 21*) were examined (Table 1, Kimura‐Kawaguchi et al., 2014). The methods used for the amplification of microsatellite DNA and genotyping of PCR products were the same as those employed in a previous study (Kimura‐Kawaguchi et al., 2014).

## 3 Results

### 3.1 Genealogical analysis of *Hexagrammos octogrammus* and the two natural hybrid strains using mtDNA

Nucleotide sequences were obtained for a total of 2,498 base pairs (bp), 994 bp of the Cyt *b* region, 918 bp of the 12S‐16S rRNA region, and 586 bp of the COI region. A total of 35 haplotypes (including subhaplotypes defined as the same arrangement of nucleotides except for a synonymous substitution) were identified in sequences from 109 individuals: 27 haplotypes from 40 *Hoc* individuals, 11 haplotypes from 31 *Hoc*/*Hag* individuals, and two haplotypes from 38 *Hoc*/*Hot* individuals (Table 2). The identified haplotypes had 63 polymorphic sites. Sequences of each haplotype and of each outgroup were deposited in GenBank under the accession numbers listed in Table S1.

**Table 2:** Variable nucleotide sites in 2,498 bp of 35 haplotypes, including three subhaplotypes in the three mtDNA regions assayed in *Hoc*,* Hoc*/*Hag*, and *Hoc*/*Hot*

Haplotypes	Nucleotide position																																																														
	*Cyt b*																																			*12S‐16S rRNA*													*COI*														
																																			1	1	1	1	1	1	1	1	1	1	1	1	1	1	1	1	2	2	2	2	2	2	2	2	2	2	2	2	2
	1	2	2	2	2	2	3	3	4	4	5	5	5	5	5	5	6	6	7	7	7	7	7	8	8	9	9	9	9	9	9	9	9	9	0	1	3	4	4	6	6	6	6	7	7	7	8	9	9	9	1	1	1	1	2	3	3	3	3	4	4	4	4
	8	2	4	4	7	8	3	4	0	5	3	4	5	6	7	9	6	8	2	3	5	6	7	8	9	0	3	3	4	4	4	6	6	9	5	8	4	1	6	4	5	6	7	0	2	8	3	2	4	8	4	7	8	9	1	0	5	7	8	0	1	3	4
	6	2	4	9	3	5	7	8	8	6	4	0	5	7	6	7	9	1	6	5	6	2	7	2	7	0	0	6	2	5	8	0	3	0	1	8	6	8	6	6	1	1	6	9	8	0	6	7	4	9	5	2	1	0	7	4	5	0	5	6	5	6	5
hap1	A	C	C	C	A	T	C	A	T	C	G	A	A	T	C	G	A	T	G	A	A	C	C	C	T	T	G	C	T	T	A	C	G	T	G	T	G	T	C	C	C	T	C	C	T	T	A	C	A	G	C	G	C	A	G	T	G	G	A	G	A	C	G
hap2	.	.	.	.	.	.	.	.	.	.	.	.	.	.	.	.	.	.	.	.	.	.	.	.	.	.	.	.	.	.	.	.	.	.	.	.	.	.	.	T	.	.	.	.	.	.	.	.	.	.	.	.	.	.	.	.	.	.	.	.	.	.	.
hap3‐1	.	.	.	.	.	.	.	G	.	.	.	.	.	.	.	A	.	.	.	.	.	.	.	T	.	.	.	.	.	.	.	T	.	.	.	C	.	.	.	.	.	.	.	.	.	.	G	.	.	A	.	A	.	.	.	.	.	.	.	.	.	.	.
hap3‐2	.	.	.	.	.	.	.	.	.	.	.	.	.	.	.	A	.	.	.	.	.	.	.	T	.	.	.	.	.	.	.	T	.	.	.	C	.	.	.	.	.	.	.	.	.	.	G	.	.	A	.	A	.	.	.	.	.	.	.	.	.	.	.
hap4	.	.	.	.	.	.	.	.	.	.	A	.	.	.	T	.	.	.	.	.	.	.	.	.	.	.	.	.	.	.	.	.	.	.	.	C	.	.	.	.	.	.	.	.	A	.	G	.	.	A	.	.	.	.	.	.	.	.	.	.	.	.	.
hap5	.	.	.	.	.	.	.	.	.	.	A	.	.	.	T	.	.	.	.	.	.	.	.	.	.	.	.	.	.	.	.	.	.	.	.	C	.	.	.	.	.	.	.	.	.	.	G	.	.	A	.	.	.	.	.	.	.	.	.	.	.	.	.
hap6	G	.	.	.	.	.	.	.	.	.	.	.	.	.	.	.	.	.	.	.	.	.	.	.	.	.	.	.	.	.	.	.	.	.	.	C	.	.	.	.	.	.	.	T	.	.	G	.	.	A	.	.	.	.	.	.	.	.	.	.	.	.	.
hap7	.	T	.	.	.	.	.	.	.	.	.	T	.	.	.	.	G	.	.	.	.	.	.	.	C	.	.	.	.	.	.	.	.	.	.	C	.	.	.	.	.	.	.	.	.	.	G	.	.	A	.	.	T	G	.	.	.	.	.	.	T	.	.
hap8	.	.	T	.	.	.	.	.	.	.	.	.	.	.	.	.	.	C	.	.	.	.	.	.	.	.	.	T	.	.	.	.	.	.	.	C	.	.	.	.	.	.	.	.	.	.	G	.	.	A	.	.	.	.	.	.	.	.	.	.	.	.	.
hap9	.	.	T	.	.	.	.	G	.	.	.	.	.	.	.	.	.	.	.	.	.	.	.	.	.	.	.	.	.	.	.	.	.	.	.	C	.	.	.	.	.	.	.	.	.	.	G	.	.	A	.	.	.	.	.	.	.	.	.	.	.	.	.
hap10	.	.	T	.	.	.	T	.	.	.	.	.	.	.	.	.	.	.	.	.	G	T	.	.	.	.	.	.	.	.	G	.	.	.	.	C	.	.	.	.	.	.	.	.	.	.	G	.	.	A	.	.	T	.	.	.	.	.	.	.	.	.	.
hap11	.	.	.	T	.	.	T	.	C	.	.	.	.	.	.	.	.	.	.	.	.	.	.	.	.	.	.	.	C	.	.	.	.	.	.	C	.	.	.	.	.	.	.	T	.	.	G	.	.	A	.	.	T	.	A	.	.	.	.	.	.	.	.
hap12	.	.	.	.	G	C	T	.	.	.	.	.	.	.	.	.	.	.	.	.	.	.	T	.	.	.	.	.	.	.	.	.	.	.	.	C	.	.	.	.	.	.	.	.	.	.	G	T	.	A	.	.	T	.	.	C	.	.	.	.	.	.	A
hap13	.	.	.	.	.	.	T	G	.	.	.	.	.	C	.	.	.	.	.	.	G	.	.	.	.	.	A	.	.	.	.	.	.	.	.	C	.	.	.	.	A	.	.	.	.	.	G	.	.	A	.	.	T	.	.	.	.	.	.	.	.	.	.
hap14	.	.	.	.	.	.	T	G	.	.	.	.	.	.	.	.	.	.	.	.	G	.	.	.	.	C	.	.	.	.	.	.	.	.	.	C	.	.	.	.	.	.	.	.	.	.	G	.	.	A	.	.	T	.	.	.	A	.	.	.	.	.	.
hap15‐1	.	.	.	.	.	.	.	C	.	.	.	.	.	.	.	.	.	.	.	.	.	.	.	.	.	.	.	.	.	.	.	.	.	.	.	C	.	.	.	.	.	.	.	.	.	.	G	.	.	A	.	.	.	.	.	.	.	.	.	.	.	.	.
hap15‐2	.	.	.	.	.	.	.	.	.	.	.	.	.	.	.	.	.	.	.	.	.	.	.	.	.	.	.	.	.	.	.	.	.	.	.	C	.	.	.	.	.	.	.	.	.	.	G	.	.	A	.	.	.	.	.	.	.	.	.	.	.	.	.
hap15‐3	.	.	.	.	.	.	.	.	.	.	.	.	.	.	.	.	.	.	.	.	.	.	.	.	.	.	.	.	.	.	.	.	.	.	.	C	.	.	.	.	.	.	.	.	C	.	G	.	.	A	.	.	.	.	.	.	.	.	.	.	.	.	.
hap16	.	.	.	.	.	.	.	.	.	T	.	.	.	.	.	.	.	.	.	.	.	.	.	.	.	.	.	.	.	.	.	.	.	.	.	C	.	.	.	.	.	.	.	.	.	.	G	.	.	A	.	.	.	.	.	.	.	.	T	.	.	.	.
hap17	.	.	.	.	.	.	.	.	.	.	A	.	.	.	.	.	.	.	.	.	.	.	.	.	.	.	.	.	.	.	.	.	.	.	.	C	.	.	.	.	.	.	.	.	.	.	G	.	.	A	.	.	.	.	.	.	.	.	.	.	.	.	.
hap18	.	.	.	.	.	.	.	.	.	.	A	.	T	.	T	.	.	.	.	.	.	.	.	.	.	.	.	.	.	.	.	.	.	.	.	C	.	.	.	.	.	.	.	.	.	.	G	.	.	A	.	.	.	.	.	.	.	A	.	.	.	.	.
hap19	.	.	.	.	.	.	.	.	.	.	A	.	.	.	T	.	.	.	.	.	.	.	.	.	.	C	.	.	.	.	.	.	.	.	.	C	A	.	.	.	.	.	.	.	.	.	G	.	.	A	.	.	.	.	.	.	.	.	.	.	.	.	.
hap20	.	.	.	.	.	.	.	.	.	.	A	.	.	.	T	.	.	.	.	G	.	.	.	.	.	.	.	.	.	C	.	.	.	.	.	C	.	.	.	.	.	.	.	.	.	.	G	.	.	A	.	.	.	.	.	.	.	.	.	.	.	.	.
hap21	.	.	.	.	.	.	.	.	.	.	.	T	.	.	.	.	.	.	.	.	.	.	.	.	.	.	.	.	.	.	.	.	.	.	.	C	.	.	.	.	.	.	.	.	.	.	G	.	.	A	T	.	.	.	.	.	.	.	.	.	.	.	.
hap22	.	.	.	.	.	.	.	.	.	.	.	.	.	.	.	A	.	.	.	.	.	.	.	T	.	.	.	.	.	.	.	T	.	.	.	C	.	.	.	.	.	.	T	.	.	.	G	.	.	A	.	A	.	.	.	.	.	.	.	A	.	.	.
hap23	.	.	.	.	.	.	.	.	.	.	.	.	.	.	.	A	.	.	.	.	.	.	.	T	.	.	.	.	.	.	.	T	.	.	.	C	.	.	.	.	.	.	T	.	.	.	G	.	.	A	.	A	.	.	.	.	.	.	.	.	.	.	.
hap24	.	.	.	.	.	.	.	.	.	.	.	.	.	.	.	A	.	.	.	.	.	.	.	T	.	.	.	.	.	.	.	T	.	.	.	C	.	.	.	.	.	.	.	.	.	.	G	.	.	A	.	.	.	.	.	.	.	.	.	.	.	.	.
hap25	.	.	.	.	.	.	.	.	.	.	.	.	.	.	.	.	.	.	A	.	.	.	.	.	.	.	.	.	.	.	.	.	.	.	.	.	.	.	.	.	.	.	.	.	.	.	.	.	.	A	.	.	.	.	.	.	.	.	.	.	.	.	.
hap26	.	.	.	.	.	.	.	.	.	.	.	.	.	.	.	.	.	.	.	.	.	.	.	.	.	.	.	.	.	.	.	T	.	.	.	C	.	.	.	.	.	.	.	.	.	.	G	.	G	A	.	.	.	.	.	.	.	.	.	.	.	.	.
hap27	.	.	.	.	.	.	.	.	.	.	.	.	.	.	.	.	.	.	.	.	.	.	.	.	.	.	.	.	.	.	.	.	A	.	.	C	.	C	.	.	.	.	.	.	.	.	G	.	.	A	.	.	.	.	.	.	.	.	.	.	.	.	.
hap28	.	.	.	.	.	.	.	.	.	.	.	.	.	.	.	.	.	.	.	.	.	.	.	.	.	.	.	.	.	.	.	.	A	.	.	C	.	.	.	.	.	.	.	.	.	.	G	.	.	A	.	.	.	.	.	.	.	.	.	.	.	.	.
hap29	.	.	.	.	.	.	.	.	.	.	.	.	.	.	.	.	.	.	.	.	.	.	.	.	.	.	.	.	.	.	.	.	.	C	A	C	.	.	.	.	.	.	.	.	.	C	G	.	.	A	.	.	.	.	.	.	.	.	.	.	.	.	.
hap30	.	.	.	.	.	.	.	.	.	.	.	.	.	.	.	.	.	.	.	.	.	.	.	.	.	.	.	.	.	.	.	.	.	.	.	C	.	.	.	.	.	.	.	.	.	.	G	.	.	A	.	.	.	.	.	.	.	.	.	.	.	T	.
hap31	.	.	.	.	.	.	.	.	.	.	.	.	.	.	.	.	.	.	.	.	.	.	.	.	.	.	.	.	.	.	.	.	.	.	.	C	.	.	A	.	.	C	.	.	.	.	G	.	.	A	.	.	.	.	.	.	.	.	.	.	.	.	.
hap32	.	.	.	.	.	.	.	.	.	.	.	.	.	.	.	.	.	.	.	.	.	.	.	.	.	.	.	.	.	.	.	.	.	.	.	C	.	.	.	.	.	.	.	.	.	.	G	.	.	A	.	.	T	.	.	.	.	.	.	.	.	.	.

Dots indicate nucleotide identity with the haplotype 1 sequence.

The maximum‐likelihood analysis showed that the two natural hybrid strains (i.e., *Hoc*/*Hag* and *Hoc*/*Hot*) formed a cluster within *Hoc*—separately from both *Hot* and *Hag*—implying that both hybrid strains had *Hoc* as the maternal species (Fig. 1). Within the *Hoc* cluster, the *Hoc*/*Hag* and *Hoc* haplotypes were mixed with the other samples in the genealogical tree, with *Hoc*/*Hag* exhibiting multiple hemiclones as if they had originated independently from separate mutations. Conversely, all of the *Hoc*/*Hot* haplotypes belonged to a single branch (Branch I) within the clade consisting of a combination and closely related *Hoc*,* Hoc*/*Hag*, and *Hoc*/*Hot* haplotypes.

**Figure 2:**
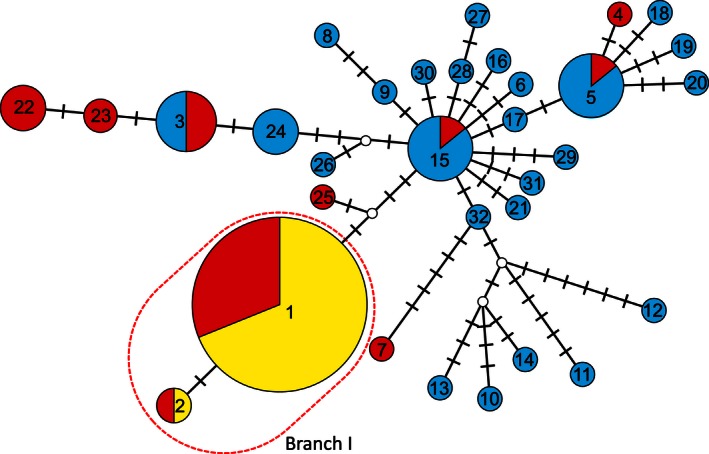
Parsimony‐based haplotype network for *Hoc*,* Hoc*/*Hag*, and *Hoc*/*Hot* inferred based on three mtDNA regions: *Cyt* b, *12S‐16S rRNA*, and *CoI*. Values in pie charts show the haplotype number. The size of each pie chart corresponds to the haplotype frequency, and the color of each pie represents *Hoc* (blue), *Hoc*/*Hag* (red), and *Hoc*/*Hot* (yellow). Open circles represent hypothetical haplotypes that were not empirically sampled. Bars indicate the synonymous substitutions required for transition between haplotypes

A total of 32 haplotypes were included in the haplotype network for *Hoc*,* Hoc*/*Hag*, and *Hoc*/*Hot* individuals (Fig. 2). Two of the haplotypes (*hap 3* and *hap 15*) contained two and three subhaplotypes (*subhap 3‐1* and *3‐2, subhap 15‐1, 15‐2* and *15‐3*), which could be distinguished from each other by a nonsynonymous substitution. From 1 to 20 mutational steps were found among the 11 *Hoc*/*Hag* haplotypes. Three of the haplotypes that *Hoc*/*Hag* shared with *Hoc* were connected by four and seven mutational steps. All 38 of the *Hoc*/*Hot* hybrids and 18 *Hoc*/*Hag* hybrids were clustered in Branch I, and 37 of the *Hoc*/*Hot* hybrids and 17 of the *Hoc*/*Hag* hybrids shared *hap 1*. A total of 56 (70.9%) of the 79 hybrids used in the present study were grouped in one cluster, and there were no haplotypes that shared the *Hoc* maternal ancestor. The extent of genetic divergence in Branch I was very low, with only one substitution in 2,498 bp detected in 12S‐16S rRNA. Conversely, the minimum number of mutational sites between *Hoc* and *Hoc*/*Hot* (*hap1* and *subhap 15‐2*) was one substitution in 12S‐16S rRNA and two substitutions in COI. Thus, the results showed that the mtDNA of *Hoc*/*Hot* was more similar to *Hoc*/*Hag* than it was to the maternal ancestor, *Hoc*.

### 3.2 Sharing of alleles among the two natural hybrid strains and *Hexagrammos octogrammus*

**Figure 3:**
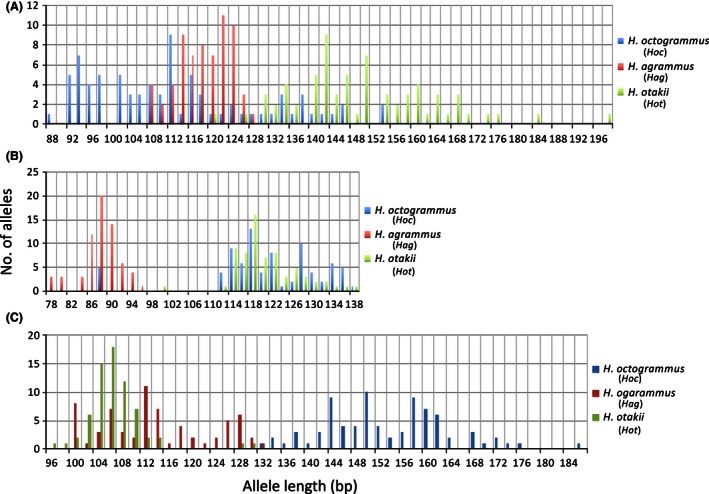
Microsatellite DNA allele frequencies in parental species used to detect the maternal alleles of hemiclonal hybrids. (A–C) Represent *hexoc 6*,* hexoc 14*, and *hexoc 21*, respectively

The characteristics of the microsatellite loci used for genotyping are shown in Table 3. In the parental species (*Hag*,* Hot*, and *Hoc*), all three loci had sufficiently high heterozygosities and low Hardy–Weinberg equilibrium deviation probabilities, which meant that the microsatellite loci were well suited for genetic analysis and that there were marked differences in the size of alleles among parental species (Fig. 3). Conversely, in the two natural hybrids, the observed heterozygosities approached to 1, except for *hexoc 6* in *Hoc*/*Hag*, and the probability of deviation from Hardy–Weinberg equilibrium assessed by a chi‐squared test was high (Table 3). This finding was illustrated by the natural hybrids that possessed a hemiclonal genome set inherited from the maternal ancestor (*Hoc*) and a different genome set inherited from the paternal species (*Hot* or *Hag*).

**Table 3:** Summary of polymorphic microsatellite loci for the two natural hybrids and the three parental species

Species name	Locus name	Size range (bp)	No of allele	*H*_o_	*H*_e_	Chi‐squared test	*p* Value
*Hexagrammos octogrammus* (*N* = 40)	*Hexoc 6*	88–154	29	0.925	0.947	0.206	.036
	*Hexoc 14*	88–138	15	0.950	0.907	0.330	.099
	*Hexoc 21*	132–186	23	0.900	0.929	0.011	.130
*Hoc*/*Hag* (*N* = 31)	*Hexoc 6*	96–128	12	0.710	0.765	0.683	.079
	*Hexoc 14*	80–134	13	0.912	0.888	0.993	.020
	*Hexoc 21*	100–162	21	1.000	0.859	0.976	.193
*Hoc*/*Hot* (*N* = 38)	*Hexoc 6*	116–182	24	1.000	0.750	1.000	1.000
	*Hexoc 14*	88–132	10	0.909	0.755	1.000	.902
	*Hexoc 21*	98–148	12	1.000	0.715	1.000	.134
*Hexagrammos agrammus* (*N* = 33)	*Hexoc 6*	108–128	11	0.921	0.883	0.868	.510
	*Hexoc 14*	78–96	9	0.818	0.812	0.025	.063
	*Hexoc 21*	100–132	17	0.879	0.909	0.889	.030
*Hexagrammos otakii* (*N* = 34)	*Hexoc 6*	120–198	25	0.941	0.938	0.347	.349
	*Hexoc 14*	100–138	15	0.853	0.877	0.927	.289
	*Hexoc 21*	96–130	12	0.824	0.828	0.259	.338

*H*_o_ and *H*_e_ exhibited observed and expected heterozygosities, respectively. The results obtained for *Hoc*,* Hoc*/*Hag*, and *Hoc*/*Hot* individuals are shown in Table 4.

Interestingly, 37 of the 38 *Hoc*/*Hot* hybrids shared the same alleles at *hexoc 6* (116 bp) and *hexoc 21* (148 bp), and all 18 of the *Hoc*/*Hag* hybrids in Branch I also shared these allele sets (Table 4). In 55 hybrids, *hexoc 14* was either 122 bp or 124 bp in length; the one exception in Branch I, ID399, shared alleles at *hexoc 14* and *hexoc 21*, but the alleles at *hexoc 6* were unique. Because the 116‐bp allele at *hexoc 6* in 37 *Hoc*/*Hot* hybrids was smaller than the size range observed in *H. otakii* (Fig. 3, Table 3), the shared allele in Branch I was likely hemiclonally inherited from the maternal ancestor, *Hoc*. The 148‐bp allele at *hexoc 21* in all 56 hybrids in Branch I was larger than the size ranges observed in both *Hot* and *Hag*, implying that the common allele was also hemiclonally inherited from *Hoc*. The 122‐bp or 124‐bp alleles at *hexoc 14* in 18 *Hoc*/*Hag* hybrids in Branch I were larger than the size range observed in *Hag*, implying that these alleles also appeared to be hemiclonally inherited from *Hoc*. The allele frequencies of the alleles (116 bp at *hexoc 6*, 122 bp or 124 bp at *hexoc 14*, and 148 bp at *hexoc 21*) that were shared among the hybrids were all less than 10% in *Hoc* (Table 3). In addition, the specific allele observed at *hexoc 6* in ID399 probably varied from homologous alleles, as microsatellite DNA occasionally mutates during generation changes (Guichoux et al., 2011). Supposing that the 122‐bp and 124‐bp alleles at *hexoc 14* were homologous and that they slipped during several generation changes, then the common homologous allele set at the three loci would occur at a frequency of less than 0.04%. Such a low rate suggested that all of the individuals in Branch I, that is, 18 *Hoc*/*Hag* and 38 *Hoc*/*Hot*, originated from the same hybridogen.

**Table 4:**
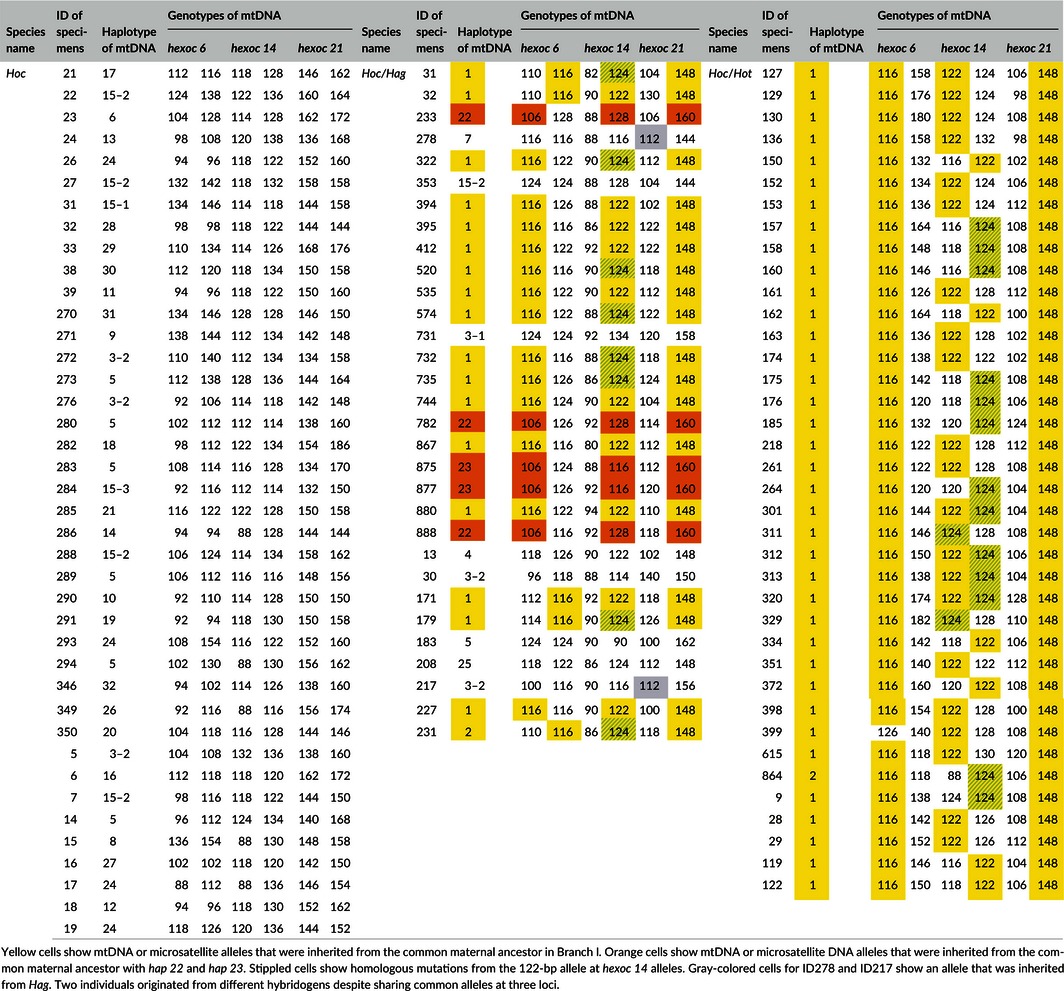
Identification (ID) number, haplotypes, and genotypes for 40 *Hoc*, 31 *Hoc*/*Hag*, and 38 *Hoc*/*Hot* fishes

Regarding the other 13 *Hoc*/*Hag* hybrids, three individuals (ID233, ID782, and ID888; all *hap 22*) shared the same alleles at the three loci examined in this study; judging from the size ranges of the alleles in *Hag* and *Hoc*, the alleles were considered to have been hemiclonally inherited from *Hoc*. Similarly, two individuals (ID875 and ID877; both *hap 23*) shared the same maternal alleles. These five individuals shared the same allele at *hexoc 6* and *hexoc 21*, even though both haplotypes had a different allele at *hexoc 14*. Only 1 bp of the 2,498 bp of mtDNA analyzed was found to differ between *hap 22* and *hap 23* (Table 2), implying that there was a high possibility that *hap 22* and *hap 23* originated from the same hybridogen.

Although ID278 (*hap 7*) and ID217 (*hap 3‐2*) shared alleles at every locus, the 112‐bp allele that was common to *hexoc 21* was inherited from *Hag*, judging from the size range of the alleles in both parental species (Fig. 3, Table 4). Of the remaining 7 *Hoc*/*Hag* hybrids, none shared any alleles at the three loci with the other hybrids.

## 4 Discussion

### 4.1 *Hoc*/*Hot* born from host switch

Because both hybridogenetic hybrids (*Hoc*/*Hot* and *Hoc*/*Hag*) had *Hoc* mtDNA haplotypes, *H. octogrammus* (*Hoc*) is considered to be the maternal ancestor of these hybrids (Crow et al., 2007; Kimura et al., 2007). Although morphological (Shinohara, 1994) and molecular studies (Crow et al., 2004) have demonstrated that *H. agrammus* (*Hag*) and *H. otakii* (*Hot*) are the closest relatives (sister species), hybrids between these two species have rarely ever been observed in areas where these species are sympatrically distributed (Crow et al., 2007; Kimura & Munehara, 2010; Kimura & Munehara, 2011). Conversely, natural hybrids (*Hoc*/*Hot* and *Hoc*/*Hag*) between distant species have been shown to propagate by hemiclonal reproduction, with hybridization occurring after secondary contact (Kimura‐Kawaguchi et al., 2014). Because hybrids typically have low fitness and survivability, parental species typically avoid hybridization by reinforcing species recognition, as failure to do so would result in these species interbreeding and forming a single species (Coyne & Orr, 2004; Ellstrand et al., 2010). Hemiclonal reproduction is one mechanism that allows hybrids to survive while avoiding genetic recombination (Burt & Trivers, 2006). Because all of the hemiclonal hybrids are fertile females capable of breeding with males of the paternal species, the two natural hybrid populations can be considered to be independent of *Hoc*,* Hot*, and *Hag* (Kimura‐Kawaguchi et al., 2014). The hybrids produce haploid eggs containing only the *Hoc* genome (maternal ancestor), as the paternal genome is discarded and F_1_ hybrid‐type offspring are generated by fertilization with haploid sperm from either *Hag* or *Hot* (paternal ancestor); the entire paternal genome is displaced at every generation change. When a *Hoc*/*Hag* hybrid mates with a *Hot* male, the entire paternal genome of the descendants will change from *Hag* to *Hot*. The genome of the descendants will therefore constitute the hemiclonal *Hoc* genome and a normal *Hot* genome, to produce the *Hoc*/*Hot* hybrids.

Genealogical analysis using mtDNA revealed that *Hoc*/*Hot* hybrids formed a cluster with *Hoc*/*Hag* (Branch 1), which did not contain any *Hoc* individuals (Fig. 1). This branch (Branch I) was supported by high bootstrap values. In the microsatellite DNA analyses, the individuals in Branch I shared a common allele set consisting of three loci, indicating that the *Hoc*/*Hot* hybrids inherited an identical hemiclonal genome set from *Hoc*/*Hag*. The low levels of diversity observed in the mtDNA and microsatellite DNA analyses showed that *Hoc*/*Hot* hybrids originated by anomalous hybridization events between *Hoc*/*Hag* and *Hot*. Although it was previously considered that the occurrence of numerous hybrids was the result of rampant hybridization between *Hoc* and *Hot* (Crow et al., 2007), *Hoc*/*Hot* hybrids are unlikely to have appeared due to interspecific hybridization. Changes in the species of the sperm donor among hybrids employing (hemi)clonal reproduction are referred to as “host switching” (Choleva, Apostolou, Rab, & Janko, 2008). Host switching has been reported in other unisexual fish lineages (e.g., *Squalius* hybrids: Alves, Coelho, Collares‐Pereira, & Dowling, 1997; *Poecilia* hybrids: Niemeitz, Kreutzfeldt, Schartl, Pazefall, & Schlupp, 2002; Schlupp, Parzefall, & Schartl, 2002; *Cobitis* hybrids: Janko et al., 2005; *Poeciliopsis* hybrids: Cunha, Coelho, Carmona, & Doadrio, 2004; Mateos & Vrijenhoek, 2002, 2005; Sousa‐Santos, Collares‐Pereira, & Almada, 2006) and amphibians (e.g., *Ambystoma* hybrids: Hedges, Bogart, & Maxon, 1992; Spolsky, Phillips, & Uzzell, 1992; *Pelophylax*: Arano, Llorente, Herrero, & Sanchiz, 1995).

Why do hybrids change the species of the sperm donor? Host switching may arise when the primary hybrids require a sperm donor after the extinction of the parental species. However, *Hexagrammos* hybrids are widespread in the North Pacific Ocean, and both paternal species (*Hag* and *Hot*) coexist. Thus, while pre‐reproductive isolation between the paternal species of this genus is likely to have occurred due to subtle differences in habitat preference and parental care (Kimura & Munehara, 2010, 2011), breeding season and site preference are known to overlap among *Hag*,* Hot*, and *Hoc* (Munehara, Takenaka, & Takenaka, 2000). For example, *Hoc* and *Hag* inhabit shallow seaweed beds, while *Hot* inhabits deeper reefs and sandy bottomed environments where seaweeds are scarce (Kimura & Munehara, 2010, 2011). *Hexagrammos* species employ breeding territories and the polyandrous females visit the multiple males' territories where they spawn and produce adhesive egg masses that are then guarded by the males (Munehara, Kanamoto, & Miura, 2000; Munehara, Takenaka, et al., 2000). Females show a preference for large males that are good guardians (Kvarnemo & Simmons, 2013; Maan & Seehausen, 2011). Because *Hot* males with territories have larger bodies and larger egg masses than *Hag* males (Munehara, Kanamoto, et al., 2000), *Hoc*/*Hag* hybrids may prefer to mate with *Hot* males. While reproductive isolation is considered to be effective for maintaining species, some anomaly must have allowed *Hoc*/*Hag* hybrids to achieve host switching at some point in the evolutionary history of these species.

In Branch I, *hap 1* was the most dominant haplotype and the difference between *hap 1* and *hap 2* was only one mutational step in 2,498 bp (Fig. 2). In addition, an identical mutation in *hexoc 14* was found in both *hap 1* and *hap 2* of *Hoc/Hag* hybrids. These findings strongly suggest that host switching first occurred as *hap 1* of *Hoc*/*Hag* became more widespread, and then *hap 1 Hoc*/*Hot* increased in number. Given that the direction of mate choice was from *Hag* to *Hot* by *Hoc*/*Hag*, and that *Hoc*/*Hot* only occurred in Branch I, the reverse host switch probably did not occur. *Hoc*/*Hag* and *Hoc*/*Hot* shared both *hap 1* and *hap 2*. It is currently not clear whether host switching occurred in *hap 2* again or not.

Thus, mtDNA and microsatellite DNA are considered to have mutated during generation changes. Assuming that the molecular clock of mtDNA in *Hexagrammos* is 1.5–2.5% per million years (Crow et al., 2004; Meyer, Kocher, Basasibwaki, & Wilson, 1990), host switching likely first occurred 17,000–27,000 years ago. Assuming a mutation rate for microsatellite DNA of 10^−3^–10^−4^ per single frameshift slippage (Guichoux et al., 2011), the first host switch occurred approximately 2,000–20,000 years ago (assuming a generation period of 2 years).

### 4.2 Diversification of *Hoc*/*Hag* from *Hoc*

**Figure 4:**
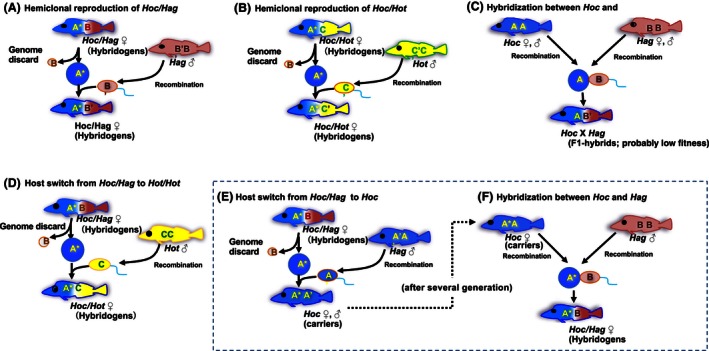
Results for hybridizations occurring among three *Hexagrammos* species and two hemiclonal hybrids. Uppercase letters superimposed on fish represent the genomes of each species, with asterisks indicating that the genome possesses the genetic factor responsible for inducing hybridogenesis. (A and B) Represent a normal backcross of hemiclonal hybrids. (C) Represents hybridization between *Hoc* and *Hag*. The F_1_ offspring (*Hoc* × *Hag*) produce gametes that have undergone recombination, but the descendants of the F_1_ offspring will disappear because genetic introgression among the parental species via two hybrid populations does not occur (Kimura‐Kawaguchi et al., 2014). (D) Represents host switching, which generated *Hoc*/*Hot*. The rectangle contains hybridization events that have not yet been observed. When a *Hoc*/*Hag* hybrid mates with a male of the maternal species, the offspring (backcrossed *Hoc*) become *Hoc* (carriers). After several generations, if these carriers mate with *Hag*, the offspring may produce a new hybridogenetic strain

There is a strong possibility that *Hoc*/*Hag* hybrids changed sperm donors to both *Hot* and *Hoc*, although the evidence is somewhat inconclusive. We consider that the genome of *Hoc*/*Hag* hybrids is constituted by both *Hoc* and *Hag* genomes, and this mode of host switching (i.e., *Hoc*/*Hag* crossing with *Hoc* instead of *Hag*) may be more likely than *Hoc*/*Hag* hybrids mating with *Hot*. When a *Hoc*/*Hag* hybrid mates with a male of the maternal species, *Hoc*, the offspring (backcrossed *Hoc*; BC‐*Hoc*) become *Hoc* (Fig. 4). BC‐*Hoc* has the same morphological characteristics as normal *Hoc*, but the two *Hoc* genomes differ somewhat with respect to the genetic material they contain. We reported previously that natural *Hoc*/*Hag* hybrids produced haploid eggs containing only the maternal genome, whereas artificial F_1_ hybrids (i.e., crosses between *Hoc* and *Hag*) produced haploid eggs containing a recombinant genome (Kimura‐Kawaguchi et al., 2014). The artificial F_1_ hybrids had the same genome composition as the natural hybrids, but the reproductive system differed between the two hybrids; that is, the *Hoc* genome of the natural hybrids carried genetic factors that facilitated hybridogenesis, which were not present in the normal *Hoc* genome. Although this mechanism has not yet been resolved at a cytological level, we found that BC‐*Hoc* individuals produced recombinant gametes (Kimura‐Kawaguchi et al., 2014; in preparation). In other words, when *Hexagrammos* species have homogeneous genomes, meiosis occurs normally in germ cells without any genome conflicts. Clonal or hemiclonal reproduction is thus one way in which the low survivability resulting from genome heterogeneity can be avoided in hybridizing organisms (Burt & Trivers, 2006; Jones & Pašakinskienė, 2005). However, hybridogenesis is very rare, occurring only when specific limitations imposed by genetic compatibility have been removed in conjunction with as yet unknown genetic factors. BC‐*Hoc* individuals can be discriminated from normal *Hoc* individuals, as the backcrosses possess genetic factors that are capable of inducing hybridogenesis. When fertile BC‐*Hoc* males mated with normal *Hoc* females, the progeny inherited the mtDNA haplotype of the normal *Hoc*. Consequently, a *Hoc* individual possessing specific genetic factors capable of inducing hybridogenesis (carrier) is considered to have altered the mtDNA haplotype. Moreover, when a carrier mates with a *Hag* male, a new hemiclone lineage will arise. Such hemiclone revival through host switching can increase the diversity of *Hoc*/*Hag* haplotypes, even if the mutation facilitating hybridogenesis may have occurred only once. Multiple haplotype revival through host switching from a single mutation in hybrids is another possible hypothesis for the observed mixing of *Hoc*/*Hag* haplotypes within the mtDNA genealogical tree.

High levels of mtDNA diversity were also found in *P. monacha‐lucida* (Quattro, Avise, & Vrijenhoek, 1991, 1992). In the *Poeciliopsis* complex, involvement of host switching through a third species (*P*. *viriosa*) appeared to generate new hemiclonal lineages (Mateos & Vrijenhoek, 2002). However, Schultz (1973) demonstrated that it was very difficult to reproduce such a clonal reproductive lineage by artificial hybridization between parental species. The intact genome of the maternal species is transferred into haploid eggs and the genome of the paternal species is eliminated. However, this means that at least two extraordinary steps must occur during oogenesis: elimination of the paternal genome and duplication of the maternal genome (Ogielska, 1994, 2009; Tunner & Heppich‐Tunner, 1991; Vinogradov, Borkin, Gunther, & Rosanov, 1990). Some of the genetic factors required for inducing hybridogenesis may be located at different loci and distributed on different chromosomes during recombination in BC‐*Hoc*. It is thus likely that only when a *Hoc* genome bearing the correct set of genetic factors hybridizes with a *Hag* genome, a new hemiclone lineage can possibly arise.

### 4.3 Improving longevity through host switching in hemiclones

In organisms that employ unisexual reproduction, individuals can produce offspring without any genetic contribution from males, and no male offspring are produced. As a result, once they arise, unisexual species are considered to be at an advantage when colonizing new habitats or when competing with sexually reproducing organisms (Avise, 2008). This theory is illustrated by the rapid expansion of Branch I in which *hap 1* was dominant (17 of 31 *Hoc*/*Hag* and 37 of 38 *Hoc*/*Hot*). Within the context of the long‐term survival of a population or species, unisexual species must mitigate the risks posed by the accumulation of deleterious mutations (Kondrashov, 1988; Welch & Meselson 2000). In hybridogenesis, the genome derived from the paternal species is renewed every generation and genetic variation is maintained; in this respect, it is different from gynogenesis in which an entire genome set is inherited by offspring. In addition, gametes are produced through recombination in sexually reproducing organisms, but not in hemiclonal systems when homologous genomes are combined. This is another advantage of hybridogenesis. Deleterious mutations that have accumulated in a hemiclone can be dispersed by recombination in carriers. Such purging of deleterious mutations is possible when hybridogens coexist with maternal species. This episodic host switching ensures that the longevity of the hemiclone lineage is improved by increasing genetic variability, provided that the maternal species continues to inhabit the hybrid zone or occurs in adjacent habitats. When did the genetic factors inducing hybridogenesis come into existence? The paternal species *Hot* and *Hag* diverged sympatrically approximately 1.2–2.0 million years ago (Crow, Munehara, & Bernardi, 2010). The mutations producing these genetic factors may possibly have arisen before speciation.

## Funding Information

This work was supported by Grants‐in‐Aid (Nos. 23380107, 26292098 and 15H02457) for Scientific Research from the Japan Society for the Promotion of Science, Japan.

## Conflict of Interest

None declared.

## Data Accessibility

Accession numbers (DDBJ) of the mtDNA sequences and the genotypes of microsatellite DNA data for specimens used in this study are shown in Table S1 and Table 4, respectively. Morphological data for specimens are provided in the supporting information for Kimura‐Kawaguchi et al. (2014).
